# Insights into Innate Sensing of Prototype Foamy Viruses in Myeloid Cells

**DOI:** 10.3390/v11121095

**Published:** 2019-11-26

**Authors:** Maïwenn Bergez, Jakob Weber, Maximilian Riess, Alexander Erdbeer, Janna Seifried, Nicole Stanke, Clara Munz, Veit Hornung, Renate König, Dirk Lindemann

**Affiliations:** 1 Host-Pathogen Interactions, Paul-Ehrlich-Institut, 63225 Langen, Germany; Maiwenn.Bergez@pei.de (M.B.); max.riess@yahoo.de (M.R.); seifriedJ@rki.de (J.S.); 2 Institute of Virology, Medical Faculty “Carl Gustav Carus”, Technische Universität Dresden, 01307 Dresden, Germany; j_weber@posteo.de (J.W.); Alexander.Erdbeer@gmx.de (A.E.); nicole.stanke@tu-dresden.de (N.S.); claramarie.munz@gmail.com (C.M.); 3 CRTD/DFG-Center for Regenerative Therapies, Technische Universität Dresden, 01307 Dresden, Germany; 4 Gene Center and Department of Biochemistry, Ludwig-Maximilians-Universität München, 81377 München, Germany; hornung@genzentrum.lmu.de; 5 German Center for Infection Research (DZIF), 63225 Langen, Germany; 6 Immunity and Pathogenesis Program, SBP Medical Discovery Institute, La Jolla, CA 92037, USA

**Keywords:** retrovirus, foamy virus, spumavirus, innate sensing, cGAS, STING

## Abstract

Foamy viruses (FVs) belong to the *Spumaretrovirinae* subfamily of retroviruses and are characterized by unique features in their replication strategy. This includes a reverse transcription (RTr) step of the packaged RNA genome late in replication, resulting in the release of particles with a fraction of them already containing an infectious viral DNA (vDNA) genome. Little is known about the immune responses against FVs in their hosts, which control infection and may be responsible for their apparent apathogenic nature. We studied the interaction of FVs with the innate immune system in myeloid cells, and characterized the viral pathogen-associated molecular patterns (PAMPs) and the cellular pattern recognition receptors and sensing pathways involved. Upon cytoplasmic access, full-length but not minimal vector genome containing FVs with active reverse transcriptase, induced an efficient innate immune response in various myeloid cells. It was dependent on cellular cGAS and STING and largely unaffected by RTr inhibition during viral entry. This suggests that RTr products, which are generated during FV morphogenesis in infected cells, and are therefore already present in FV particles taken up by immune cells, are the main PAMPs of FVs with full-length genomes sensed in a cGAS and STING-dependent manner by the innate immune system in host cells of the myeloid lineage.

## 1. Introduction

Spuma or foamy viruses (FVs), which constitute several genera in the retrovirus subfamily *Spumaretrovirinae* [1], display a replication strategy with features common to both other retroviruses (*Orthoretrovirinae*) and hepadnaviruses (reviewed in [2,3]). FVs are unique amongst retroviruses, as the initiation of reverse transcription (RTr) of the packaged viral genomic RNA (vgRNA) occurs in a significant fraction of virions (5–10%) during viral assembly [4,5,6,7]. Thereby, unlike to orthoretroviruses, both vgRNA and/or viral genomic DNA (vgDNA) containing virions are found in the supernatant of FV infected cells. It is generally accepted that the vgDNA containing virions contribute to the majority of new productive infection events during spreading of FVs in cultures, at least in vitro [5,6]. However, a low level of reverse transcription, probably derived from vgRNA containing virions, has been observed during uptake of FVs at very low multiplicities of infection (MOI) [4,7].

FVs are naturally endemic to most non-human primates (NHPs), including New and Old World monkeys and apes, cats, cows, horses, tree shrews, sea lions, and bats (reviewed in [2,8,9]). In addition, endogenized copies of FV genomes were identified in sloths, the aye-aye, the Cap golden mole [10,11], cod [12], platyfish [12], zebra fish, and the coelacanth. Nowadays, humans are not considered as a natural host, but frequent zoonotic transmissions of NHP simian FVs (SFVs), but not feline FV (FFV) or bovine FV (BFV), have been observed in workers occupationally exposed to NHPs—bush meat hunters in central Africa, and in various contexts of human–NHP interspecies contact in South and Southeast Asia. Cases of spread from human to human have not been reported. The best-studied and characterized isolate to date is the so-called prototype FV (PFV; formerly known as human FV, HFV), which was originally isolated from an African patient who presumably was infected zoonotically by a chimpanzee FV [13,14,15].

Another characteristic of FVs is their extremely broad tropism. In vitro, only very few species and cell types are known to be non-permissive to FVs or FV Env-mediated entry [16]. FV infection in vitro is highly cytopathic to most cell types, except cell lines or primary cells of myeloid or lymphoid origin, which can become chronically infected [17,18]. The cellular targets of FVs in vivo remain poorly characterized. In infected monkeys, the viral genome is detectable in many tissues but appears to be largely in a latent state, as viral replication is reported to be mainly restricted to the superficial epithelial layer of the oral mucosa [19]. This explains the major transmission mode between monkeys and zoonosis to humans through bites. In the blood, proviral DNA is detected in CD8^+^- and CD4^+^ T-cells (memory and naïve) as well as B-cells, and to a lower extent also in monocytes and NK-cells [20,21,22]. Other types of immune cells that encounter FVs during the course of an infection in vivo have not been characterized.

FVs have co-evolved with their hosts for at least 60 million years and are considered to be non-pathogenic in natural hosts and zoonotically infected humans. The immune system seems to control FV infection very efficiently in natural hosts and zoonotically infected humans, as replication and viral load of FV stays low [20,21,22]. However, the mechanisms involved are poorly understood (reviewed in [8]). In particular, the interaction of FV with the innate immune system remains to be fully clarified. FVs are known to respond in vitro in a cell type-dependent manner towards IFNs [23]. Furthermore, treatment of cells of different origin with type-I or II IFNs, impairs spreading of FVs or inhibits early steps in FV replication [24,25,26,27,28]. In line with this observation, restriction of FVs by several IFN-induced cellular proteins has been reported, although their relevance for FV replication in vivo has not been tested so far [29,30,31,32]. Although FV replication in vitro is impaired by addition of exogenous IFNs, infection of a variety of tissues does not seem to mount an innate immune response [28]. Only recently, stimulation of IFN secretion by human hematopoietic cells, in particular, plasmacytoid dendritic cells (pDCs), upon incubation with SFV virions or SFV infected cells, was demonstrated [23]. pDCs, as the main producer of type-I IFN, detect FV RNA by the TLR7-mediated pathway. However, the contribution of myeloid cells, besides pDCs, to innate sensing and IFN induction has as of yet not been investigated. Above all, conventional dendritic cells (DCs) play a critical role in detecting retroviruses, as shown for the lentivirus HIV-1 [33], and promote, thereby, the activation of adaptive immunity [34]. For HIV-1 and other lentiviruses, it has been demonstrated that the reverse transcribed DNA products generated upon viral entry in DCs mediate the activation of transcription factors, such as IRF3, resulting in ISG expression and IFN synthesis [33,35]. Thereby, the viral reverse transcribed components are sensed by the DNA sensor cyclic GAMP synthase (cGAS) [36] together with polyglutamine binding protein 1 (PQBP1) [37]. Interestingly, and in contrast to lentiviruses, FV infected cells release both vgRNA and vgDNA containing virions, and harbor RTr products late during the assembly steps. Therefore, exposure of DCs and other myeloid cells to FVs may result in interactions with the innate immune system different to those of other retroviruses. For instance, they could include pathways encountered by hepatitis B virus (HBV), as HBVs, like FVs, reverse transcribe their genome during particle morphogenesis (reviewed in [38]).

The aim of this study was to investigate whether the innate immune system of cells of the human myeloid lineage is capable of sensing FVs. If so, the viral pathogen-associated molecular patterns (PAMPs), the pattern recognition receptors (PRRs), and sensing pathways involved were to be characterized. We found that the innate immune system responds to sensing of RTr products of full-length PFV genomes but not of minimal vector genomes in the cytoplasm of human myeloid cells with an efficient interferon-stimulated gene (ISG) induction within hours of virus exposure. Sensing of PFV RTr products was dependent on cellular cGAS and STING expression and largely insensitive to reverse transcriptase inhibition during viral entry, suggesting that the already viral DNA (vDNA) containing PFV particles are the main stimulator.

## 2. Materials and Methods

### 2.1. Cells and Culture Conditions

The human embryonic kidney cell line 293T (ATCC CRL-1573) [39] and a proteoglycan-deficient variant 293T-25A (described elsewhere), the human fibrosarcoma cell line HT1080 (ATCC CCL-121) [40], and the clonal variant HT1080 PLNE containing a PFV LTR driven *EGFP* reporter gene expression cassette [41], were cultivated in Dulbecco’s modified Eagle’s medium (DMEM) supplemented with 10% (*v*/*v*) heat-inactivated fetal calf serum and antibiotics. The human monocyte cell line THP-1 (ATCC TIB-202) and knockout (KO) variants ∆IFNAR1, ∆SAMHD1 [42], ∆cGAS [43], ∆MAVS [43], ∆MyD88, and ∆STING [43] were cultivated in Roswell Park Memorial Institute 1640 Medium (RPMI 1640) supplemented with 10% (*v*/*v*) heat-inactivated fetal calf serum, antibiotics, 2.5 g/L glucose, 10 mM Hepes, and 10 mM sodium pyruvate. THP-1 cells were differentiated into macrophage-like cells by the addition of phorbol-12-myristyl-13-acetate (PMA) at 30 to 50 ng/mL final concentrations for 48 h prior to exposure of viral supernatants.

Human buffy coats, of anonymous blood donors, were obtained from German Red Cross Blood Donor Service Baden–Württemberg Hessen. Primary human monocytes were isolated from peripheral blood mononuclear cells (PBMCs) using Ficoll density gradient and subsequent isolation of CD14^+^ monocytes [44]. Briefly, PBMCs were separated by density gradient centrifugation (30 min, 980× *g*, RT), using Ficoll (Histopaque 1077 Sigma-Aldrich Biochemie GmbH, Hamburg, Germany). The mononuclear layer of the interphase was recovered and washed twice with PBS. PBMCs were subsequently incubated with 0.86% (*w*/*v*) ammonium chloride for erythrocyte lysis (37 °C, 10 min). PBMCs were washed twice and filtered (0.7 µm). CD14^+^ monocytes were positively selected using CD14-MicroBeads (Miltenyi Biotec, Bergisch Gladbach, Germany) according to the manufacturer’s protocol, and separated from unlabeled cells using an AutoMACS device (Miltenyi Biotec). Subsequently, cells were differentiated into monocyte derived dendritic cells (MDDC) or monocyte derived macrophages (MDMs) by cultivating for 5 days in RPMI-1640 medium supplemented with 2 mM l-Glutamine; 10% (*v*/*v*) FCS; 1% (*v*/*v*) HEPES, 1 mM sodium pyruvate; 280 U/mL granulocyte-macrophage colony-stimulating factor (GM-CSF) (Leukine^®^ Sargramostim, Genzyme, Boston, MA, USA); 800 U/mL IL-4 (PeproTech GmbH, Hamburg, Germany) for MDDCs and 560 IU/mL GM-CSF only for type 1 proinflammatory MDMs. The same amounts of medium and cytokines were added on day 3. On day 5 of differentiation, MDMs were detached by a short incubation with PBS-EDTA and MDDCs by gentle resuspension in PBS; they were counted and plated for cell stimulation experiments and simultaneously checked for MDDC and MDM surface markers and their differentiation status.

### 2.2. Recombinant Plasmid DNAs

A four-component PFV vector system, consisting of the expression-optimized packaging constructs pcoPG4 (PFV Gag), pcoPE (PFV Env), and pcoPP (Pol), and an enhanced green fluorescent protein (EGFP)-expressing PFV transfer vector pMD9 or puc2MD9, has been described previously [16,45,46].

The CMV-driven proviral expression vector pczHSRV2 (wt) and its variants pczHSRV2 M69 (iRT), expressing a Pol protein with enzymatically inactive RT domain (YVDD_312–315_GAAA mutation), pczHSRV2 M73 (iIN), with enzymatically inactive IN domain (D_936_A mutation), and pczHSRV2 EM271 (∆Env), with inactivated Env translation start (M1T, ATG to ACG, M5T, ATG to ACG; M16T, ATG to ACG mutation) were described previously [5,47,48]. For this study the variants pczHSRV2 EM284 (∆Gag), with inactivated Gag translation start (M1L, ATG to CTG; S3Stop, TCA to TAA mutation); pczHSRV2 EM270 (∆Pol), with inactivated Pol translation start (M1L, ATG to CTG mutation); pczHSRV2 EM273 (∆GPE), with simultaneously inactivated Gag, Pol, and Env translation starts; pczHSRV2 EM020 (iFuse), with inactivated Env SU/TM furin cleavage site (R571T mutation); and pczHSRV2 EM010, with inactivated Tas translation start (M1L, ATG to TTG mutation), were generated. All constructs were verified by sequencing analysis. Primer sequences and additional details are available upon request.

For VLP-Vpx production the lentiviral vector pSIV3+, derived from SIVmac251 was used, as previously described [49]. For single-round HIV-1 reporter virus production the plasmid pBR-NL43-Env^−^-IRES-eGFP-nef^+^ [50] was used. In both cases the envelope vector pCMV-VSVg was used for pseudotyping.

### 2.3. Transfection, Virus Production, and Titration

Cell culture supernatants containing recombinant PFV particles and respective mock controls were generated by transfection of the corresponding virus encoding or mock control plasmids (mock A: pUC19; mock B: pczHSRV2 EM273 (∆GPE)) into 293T cells using polyethyleneimine (PEI) as described previously [41,46], or 293T-25A cells using calcium phosphate (described elsewhere). Cell-free supernatants were generated by passing through a 0.45 µm filter and were either stored in aliquots at −80 °C when used for stimulation experiments or processed further for additional analysis. Viral titers of recombinant, EGFP-expressing PFV vector particles (PFV-SRVs) by fluorescence marker-gene transfer assay on HT1080 cells were determined as described previously [51]. Virus particles generated by use of proviral expression plasmids (PFV-RCPs) were titrated on HT1080 PLNE cells harboring a Tas-inducible nuclear *EGFP* ORF in their genome as described previously [41].

VLP-Vpx and HIV-1 GFP reporter viruses were produced as previously described [52]. Briefly, 2 × 10^7^ HEK293T/17 cells per T175 flask were seeded. The next day, 15.2 µg pSIV3+ and 2.3 µg pCMV-VSVg for VLP-Vpx production and 11.6 µg of pBR-NL43-Env^−^-IRES-eGFP-nef^+^ and 5.9 µg pCMV-VSVg, for HIV-1 reporter virus production, per flask, were transfected using 18 mM PEI (Sigma-Aldrich). Medium was changed approximately 16 h later and viral supernatants were harvested 48 and 72 h post-transfection. Supernatants were centrifuged (10 min at 4 °C; 1500 rpm), filtered (0.45 µm), and DNaseI digested (1 U/mL) for one hour. Viral supernatants were purified by ultracentrifugation through 20% (*w*/*v*) sucrose (2 h, at 4 °C; 25,000 rpm); virus pellets from day one and two were resuspended in PBS, pooled, aliquoted and stored at −80 °C. HIV-1 reporter viruses were titrated via serial dilutions on TZM-bl reporter cells using the beta-galactosidase colorimetric assay. VLP-Vpx were titrated according to their ability to target the restriction factor SAMHD1 for degradation, using Western blot.

### 2.4. Myeloid Cell Stimulation and qPCR Analysis of ISG Induction

For stimulation experiments, THP-1 cells were plated at 2 × 10^6^ cells/well (3 × 10^4^ cells/well) in a total volume of 2 mL (100 µL) in 6-well (96-well) plates and PMA was added to 30 ng/mL (50 ng/mL) final concentration. Forty-eight hours later the medium was replaced by the respective virus supernatant, mock control supernatants (mock A: pUC19, mock B: PFV-RCP ∆GPE mutant) or medium (medium) as indicated. At different time points post virus exposure, as indicated, viral supernatants were aspirated and cells were snap frozen at −80 °C and stored until subsequent nucleic acid extraction. MDDCs and MDMs were plated at 3 × 10^4^ cells/well in the differentiation medium without cytokines, 24 h prior to infection. Virus supernatants, either PFV or a VSV-G pseudotyped full-length HIV-1 GFP reporter virus, with or without the addition of VLP-Vpx were added. AZT (Sigma) was added during infection in the indicated experiment at a final concentration of 100 µM. MDDC and MDM infections were conducted by spinoculation at 1200 rpm, 32 °C for 1.5 h. Viral supernatants were replaced by fresh medium and cells were cultivated at 37 °C, 5% CO_2_. At different time points post infection, cells were either lysed for RNA extraction and subsequent RT-qPCR or stained for FACS analysis.

### 2.5. Quantitative PCR Analysis

Cellular nucleic acids from cultures in 6-well plates were extracted using the RNeasy Mini kit (QIAGEN, Hilden, Germany) according to the manufacturer’s protocol. qPCR analysis of cellular mRNA expression using Maxima Probe qPCR Master Mix including ROX dye (ThermoFisher Scientific, Dreieich, Germany), a StepOnePlus (Applied Biosystems, Foster City, CA, USA) quantitative PCR machine, and plasmid standard curves was performed as previously described [48]. Primers, Taqman probes, and cycling conditions are summarized in Table A1. Cellular nucleic acids from cultures in 96-well plates were extracted using NucleoSpin^®^ RNA Plus Kit (Macherey-Nagel, Düren, Germany) according to the manufacturer’s protocol. Relative expression levels of *ISG54* and *RPL13A* were determined using QuantiTect SYBR Green RT-qPCR Kit (QIAGEN) with the respective specific primers on a LightCycler^®^ 480 Instrument (Roche, Basel, Switzerland). Relative mRNA expression levels were normalized to the housekeeping gene *RPL13A* and analyzed using the 2^(−∆∆CT) method, finally depicted as fold inductions over mock A, mock B, or medium, as indicated. Primers and cycling conditions are summarized in Table A2. Primer efficiencies have been tested before in 10-fold serial dilutions and were calculated to have >90% efficiency.

### 2.6. Flow Cytometry Analysis

Purity of MDMs and MDDCs was assessed via flow cytometry analysis. Triple stainings, of 1 × 10^5^ cells with CD14-Pacific blue (BioLegend, San Diego, CA, USA), CD163-PE (BD), CD206-APC (BD) and CD1a-PE (BioLegend), and CD11c-Vio Blue (BioLegend) and CD16-APC (BioLegend) were performed with the matching IgG controls, listed in Table A3. In order to determine CD86 activation, marker expression upon infection with different PFV mutants, 24 h post infection, 6 × 10^4^ cells were stained with CD86-PE (Biolegend) or the corresponding isotype control. Briefly, after 5 days of differentiation, MDMs were detached by a short incubation with PBS-EDTA, MDDCs by gentle resuspension in PBS. Cells were washed twice with FACS staining buffer (PBS containing 10% (*v*/*v*) FCS), FC-Block (1:10, BD) was added to prevent unspecific binding via Fc-receptors (10 min, RT), cells were resuspended in the specific antibody dilutions or corresponding isotype-controls, and they were stained for 20 min on ice. Subsequently, cells were washed twice with FACS staining buffer, fixed with ice-cold 2% (*w*/*v*) Paraformaldehyde, washed twice, and analyzed via flow cytometry using MACSQuant Analyzer 10 (Miltenyi) and FCS Express software (De Novo Software, Glendale, CA, USA). For MDM and MDDC purity, percentages of positive cells of the specific markers are depicted in Figure S1. For CD86 expression of infected MDDCs, mean fluorescent intensities (MFIs) normalized to the IgG controls of each condition are depicted as relative MFIs normalized to the wt treatment. Infection levels by HIV-1 GFP with and without AZT were assessed by determining the percentage of GFP-positive cells (Figure S2). The cut-off was set to 0.1% with the IgG controls or the non-infected controls.

### 2.7. Analysis of PFV Particle Protein and Nucleic Acid Composition

PFV particles were concentrated from cell-free supernatants by centrifugation at 4 °C and 25,000 rpm for 3 h in a SW32Ti rotor (Beckman Coulter GmbH, Krefeld, Germany) through a 20% (*w*/*v*) sucrose cushion. The particulate material was resuspended in phosphate-buffered saline (PBS) and used immediately for further analysis or stored at −80 °C. Western blot analysis was performed after protein sample buffer addition as described before using PFV Gag and PFV Env leader peptide (LP) specific antisera [48]. DNase digestion of viral particles, extraction of particle-associated nucleic acids, and analysis of nucleic acid composition by qPCR was done using the primer–probe sets listed in Table A1, as described before [46,48,53].

### 2.8. Analysis of Cellular Protein Expression

For immunoblot analysis of IRF3 phosphorylation and SAMHD1 degradation, 5 × 10^5^ MDDCs/12 wells were seeded and exposed to either PFV-RCP or VLP-Vpx. Six or twenty-four hours post-exposure, cells were harvested by resuspension in ice cold PBS, centrifuged (300× *g*, 6 min, 4 °C), and lysed in 25 µL RIPA-lysis buffer (100 mM NaCl; 10 mM EDTA (pH 7.5), 20 mM Tris (pH 7.5); 1% (*v*/*v*) Triton X-100; 1% (*w*/*v*) sodium deoxycholate) containing protease and phosphatase inhibitor cocktails (Complete Protease Inhibitor Cocktail; PhosSTOP Phosphatase Inhibitor Cocktail, Roche) for 45 min on ice. Lysates were centrifuged (17,000× *g* for 15 min at 4 °C), and protein concentration was determined based on the Bradford assay using the Bio-Rad Protein Assay Dye Reagent Concentrate. Samples containing 20 µg protein were prepared with NuPAGE LDS sample buffer (4×) and NuPAGE Sample Reducing Agent (10×), to a final 1× concentration and denatured at 70 °C for 10 min. Proteins were separated on precasted NuPAGE™ 4–12% Bis-Tris gradient gels (Invitrogen). The gel was run in 1× MOPS buffer (1 M MOPS, 1 M Tris, 69.3 mM SDS, 20.5 mM EDTA Titriplex II) supplemented with 200 μL NuPage Antioxidant 10× (inner chamber) at 200 V for 1 h 10 min. Proteins were transferred to a Hybond P 0.45 PVDF membrane (GE Healthcare, Chicago, IL, USA) using the XCell IITM blotting system with 1× NuPAGE transfer buffer (Invitrogen) at 35 V for 1 h 40 min. Membranes were blocked in 5% (*w*/*v*) BSA (Carl Roth) in 0.01% (*v*/*v*) Tris-buffered saline with Tween 20 (TBST) for 2 h at 4 °C with subsequent incubation in primary antibody dilutions at 4 °C overnight. Horseradish peroxidase (HRP)-linked goat anti-rabbit or horse anti-mouse IgG (heavy and light chain) secondary antibodies (Cell signaling, Danvers, MA, USA) were applied for 2 h at 4 °C. For detection Pierce^®^ ECL Western Blotting Substrate (ThermoFisher Scientific) or ECL Prime (GE Healthcare) were used and the emitted chemiluminescence was detected at different exposure times on autoradiography films (Amersham Hyperfilm ECL, GE Healthcare). The following primary antibodies were used and applied at 4 °C, overnight: Anti-Phospho-IRF-3 (Ser396) (Cell Signaling, number 4947); Anti-IRF3 (Epitomics, number 2241-1); Anti-GAPDH (Cell Signaling, number 2118); and Anti-SAMHD1 (Proteintech; number 12586-1-AP). In order to remove phospho-IRF3 antibody, probed membrane was incubated in stripping buffer (2% (*w*/*v*) SDS, 62.5 mM Tris-HCl (pH 6.8), 100 mM β-mercaptoethanol), rotating for 45 min at 65 °C.

### 2.9. Statistics

All the statistical analyses were performed using GraphPad Prism 8. The numbers of experimental replicates and information on the statistical methods used for determination of two-tailed *p*-values are described in the individual figure legends. Symbols represent: * *p* < 0.05; ** *p* < 0.01; *** *p* < 0.001; **** *p* < 0.0001; ns: not significant (*p* ≥ 0.05).

## 3. Results

### 3.1. ISG Induction in Myeloid Cells upon Exposure to Replication-Competent PFV

**Figure 1:**
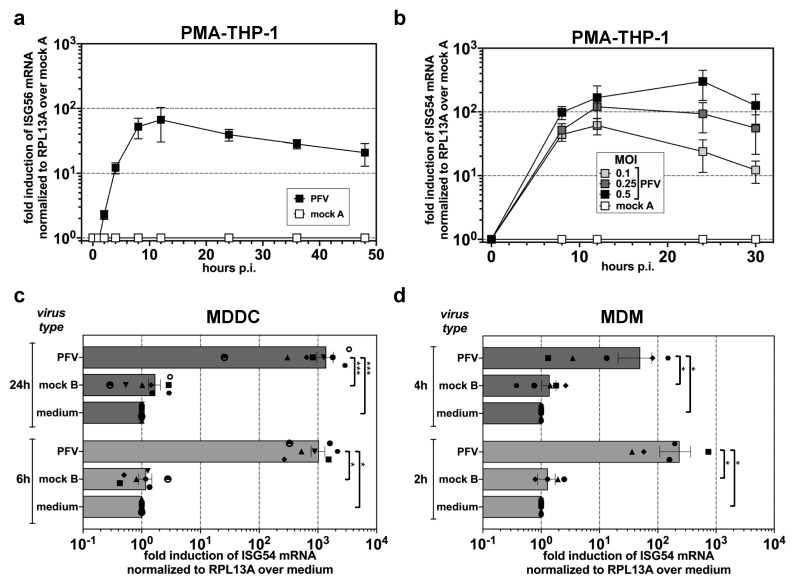
PFV-mediated ISG induction in myeloid cells. (**a**,**b**) Kinetics of *ISG56*/*ISG54* induction in PMA-differentiated THP-1 wild type cells incubated with different amounts of wild type PFV-RCP (a, MOI 0.2) as well as pUC19 (mock A) mock supernatants. ISG mRNA levels normalized for *RPL13A* mRNA levels were determined by qPCR at the indicated time points post exposure. Means ± SDs of *ISG56* (*n* = 4; a) or *ISG54* (*n* = 4; b) induction relative to mock A treatment are shown. (**c**,**d**) Primary human MDDC (**c**) or MDM (**d**) were incubated with wild type PFV-RCP (PFV; MOI 0.25), ∆GPE (mock B) mock supernatant, or medium (medium) for 6, 12, or 24 h, as indicated. Means ± SEMs, plus individual data points, of *ISG54* (*n* = 5–8) induction normalized to *RPL13A* relative to medium treatment are shown. Mixed-effects analysis with Holm–Sidak’s multiple-comparisons test was used to assess significance. * *p* < 0.05; ** *p* < 0.01; *** *p* < 0.001; **** *p* < 0.0001; ns: not significant (*p* ≥ 0.05).

**Figure A1:**
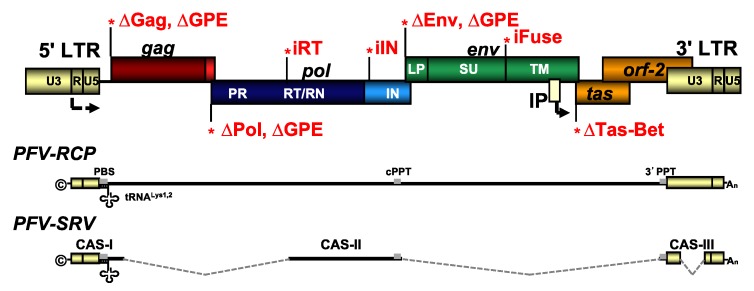
PFV genome organization and viral expression systems used. Schematic outline of the wild type (wt) PFV proviral genome structure with *gag*, *pol*, and *env* ORFs. The locations of the point mutations for the individual proviral mutants resulting in variants with enzymatically inactive integrase (iIN) or reverse transcriptase (iRT); fusion-deficient Env (iFuse); or deficiencies in the translation of, Tas (∆Tas-Bet); Gag (∆Gag); Pol (∆Pol); Env (∆Env); or Gag, Pol, and Env (∆GPE), due to inactivation of the respective translation initiation sites. PFV-RCP supernatants contain full-length viral genomic RNA (vgRNA) whereas PFV-SRV supernatants harbor vgRNA comprising the minimal essential cis-acting viral sequences (CAS). LTR: long terminal repeat; U3: unique 3′ LTR region; U5: unique 5′ LTR region; R: repeat LTR region; PR: protease; RT/RN: reverse transcriptase—RNase H; IN: integrase; LP leader peptide; SU: surface subunit; TM: transmembrane subunit; IP: internal promoter; ©: cap; A_n_: poly-A tail; PBS: primer binding site; 3′ PPT: 3′ poly purine tract; cPPT: central PPT.

PMA-differentiated THP-1 monocytic cells represent an in vitro model system recapitulating the functional properties of macrophages and dendritic cells exposed to retroviruses [54]. In order to analyze whether FVs are sensed by cells of the myeloid lineage, replication-competent PFV supernatants derived from full-length, wild type proviral expression constructs (PFV-RCP) (Figure A1) were first used to infect PMA-differentiated THP-1 cells (Figure 1a,b). Relative transcription levels of *ISG54* or *ISG56* were determined as readouts for an IRF-3 dependent stimulation, since the selected ISGs are directly downstream transcriptional targets of IRF3 [55,56].

Exposure of PMA-differentiated THP-1 cells to PFV-RCPs led to a strong IRF3-dependent ISG induction (Figure 1a,b). Furthermore, a significant, dose-dependent ISG induction was detectable, which peaked at 8 to 12 h and declined slowly thereafter (Figure 1a,b). To corroborate this finding, primary human MDMs and MDDCs were analyzed. As it cannot be ruled out that DCs and macrophages may possess slightly different sets of proteins aiding to sense DNA, we exposed both cell types to wild type PFV-RCP (PFV), mock (mock B) supernatants obtained after 293T transfection of a proviral expression construct with inactivated viral structural protein expression (∆GPE), or medium (medium). Interestingly, in both MDDCs and MDMs we detected a robust and high ISG induction (Figure 1c,d) after PFV-RCP but not mock supernatant exposure, suggesting that replication-competent PFV derived from full-length proviral expression constructs are efficiently sensed by the innate immune system in different myeloid cell types. In line with this, a strong phosphorylation of IRF3 was detectable in PFV-RCP treated but not in medium treated MDDCs at 6 h post exposure (Figure S3).

### 3.2. PFV is Sensed by the Cellular cGAS-STING Pathway

**Figure 2:**
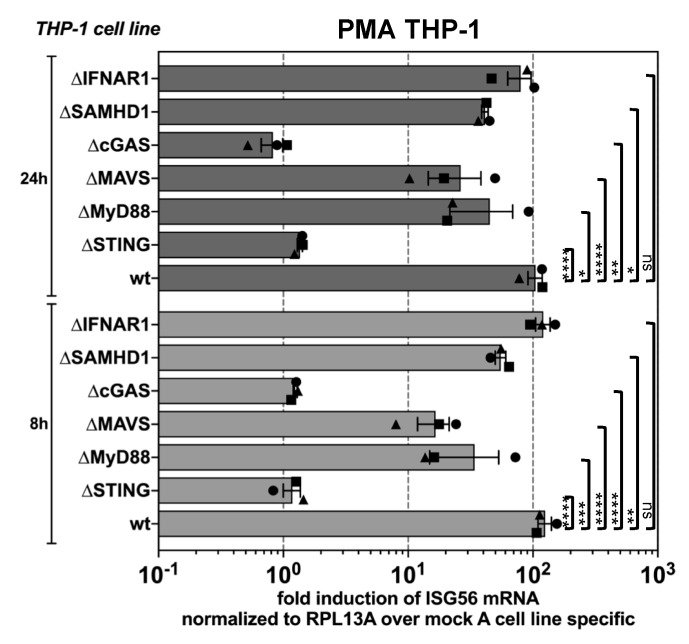
cGAS and STING-mediated sensing of PFV-associated reverse transcription products. PMA-differentiated THP-1 wild type cells and KO variants with deficiencies in components of various innate sensing pathways, as indicated, were incubated with wild type PFV-RCP (MOI 0.2) or pUC19 (mock A) mock supernatants. ISG mRNA levels normalized for *RPL13A* mRNA levels were determined by qPCR at the indicated time points post exposure. Means ± SEM, plus individual data points, of *ISG56* (*n* = 3) induction normalized to *RPL13A* relative to mock A treatment are shown. Two-way ANOVA with Tukey’s multiple-comparisons test was used to assess significance. * *p* < 0.05; ** *p* < 0.01; *** *p* < 0.001; **** *p* < 0.0001; ns: not significant (*p* ≥ 0.05).

To identify the particular innate pathways that are triggered by PFV, we exposed a panel of THP-1 KO cell lines deficient in key molecules of various sensing pathways to wild type PFV-RCP for 8 or 24 h, respectively. Interestingly, PMA-THP-1 cells deficient in cGAS or STING expression, which are key molecules of the DNA-sensing pathway, failed to mount any measurable ISG-response upon PFV exposure (Figure 2). In contrast, cells deficient in MAVS, a key node of the RIG-I/MAVS RNA-sensing pathway, or MyD88 that lies downstream of the endosomal TLR7/9 pathway showed a reduced but clearly detectable ISG induction. These results suggest that FVs are mainly sensed by the cytosolic DNA-sensing pathway in myeloid cells. Furthermore, IFNAR1 deficient PMA-THP-1 cells displayed a similar ISG response as wild type cells indicating that events downstream of IFN production do not influence sensing of FV. Intriguingly, KO of SAMHD1, previously demonstrated to strongly enhance HIV-1 sensing in various myeloid cell types [33,36,37], did not further stimulate the ISG response upon PFV exposure. On the contrary, PFV-mediated ISG induction in SAMHD1 KO cells was moderately reduced, at levels comparable to those observed for MAVS and MyD88 KO cells.

Thus, innate sensing of PFV in host cells of the myeloid lineage occurs mainly in a cGAS and STING dependent manner.

### 3.3. Innate Sensing of PFV Requires Cytoplasmic Access and Enzymatically Active Reverse Transcriptase

**Figure 3:**
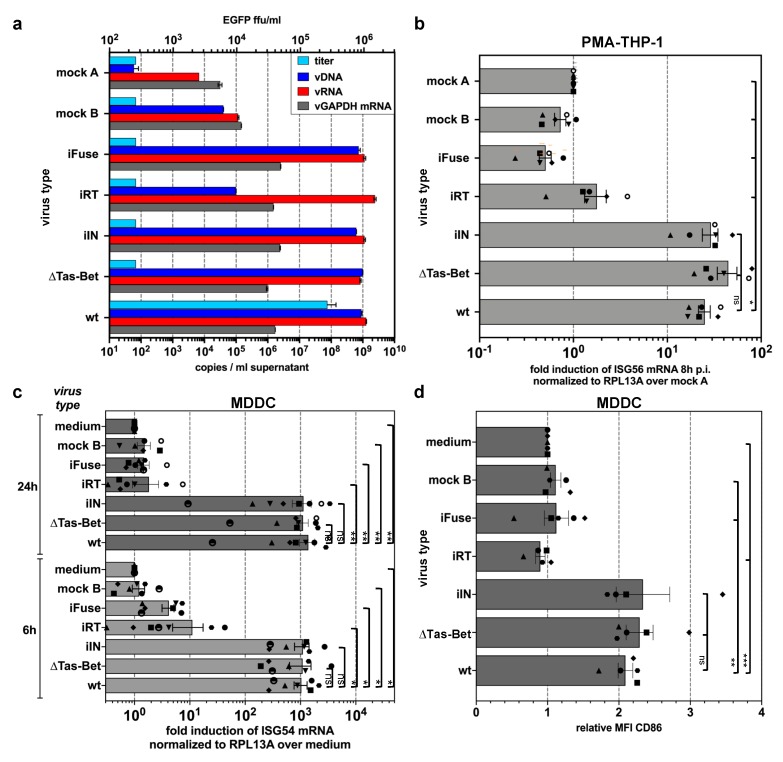
Differential ISG induction profiles of PFV mutants (described in detail in Figure A1 and Material and Methods) varying in their protein and nucleic acid composition. (**a**) Particle-associated nucleic acids extracted from viral particles pelleted by ultracentrifugation of virus supernatants used in (**b**–**d**) were analyzed by qPCR to quantify the particle-associated viral (vDNA: viral DNA; vRNA: viral RNA) and cellular (vGAPDH mRNA) nucleic acid composition. Mean copy numbers ± SDs per mL supernatant determined from duplicates are shown. (**b**,**c**) ISG induction profile of PMA-differentiated THP-1 wild type cells (**b**) or MDDCs (**c**) incubated with identical amounts of wild type PFV-RCP (MOI 0.1 THP-1; MOI 0.25 MDDCs) supernatants, variants thereof, and pUC19 (mock A) and ∆GPE (mock B) mock supernatants, or medium, as indicated, for 8 h and 24 h. Means ± SEMs, plus individual data points, of *ISG56* (*n* = 6) or *ISG54* (*n* = 5–7) mRNA induction normalized to *RPL13A* mRNA relative to mock A or medium treatment are shown. One-way ANOVA with Tukey’s multiple-comparisons test (**b**) or mixed-effects analysis with Tukey’s multiple-comparisons test (**c**) was used to assess significance. (**d**) CD86 cell surface expression profile of MDDCs 24 h post exposure to wild type PFV-RCP (MOI 0.25) supernatants and variants thereof as indicated. Means ± SEMs (*n* = 5), plus individual data points, relative to medium treatment are shown. Mixed-effects analysis with Tukey’s multiple-comparisons test was used to assess significance. * *p* < 0.05; ** *p* < 0.01; *** *p* < 0.001; **** *p* < 0.0001; ns: not significant (*p* ≥ 0.05).

Since we determined the cytoplasmic DNA-sensing pathway as the main innate pathway, we aimed at identifying the PAMPs responsible of PFV sensing and at determining the cellular sublocation. For this purpose, myeloid target cells were exposed to viral supernatants derived from various mutant proviral expression constructs, which varied in their nucleic acid compositions and had different blocks in early steps of viral replication (Figure A1, Figure 3a). A significant ISG induction was detectable in PMA-THP-1 and MDDCs not only by wild type PFV-RCP, but also by variants that either failed to express the PFV transactivator Tas and accessory protein Bet (∆Tas-Bet) after proviral integration due to inactivation of the translation start site of the *tas* ORF, or encoded an enzymatically inactive integrase (iIN) and were, therefore, largely integration-deficient (Figure 3b,c). This indicates that FV sensing does not require proviral integration or de novo viral transcription capacity.

In contrast, ISG induction was strongly reduced or undetectable in cells exposed to supernatants of PFV-RCPs either encoding an enzymatically inactive reverse transcriptase (iRT) or a fusion-deficient envelope glycoprotein (iFuse); and a control supernatant derived from a proviral expression construct with simultaneous inactivation of *gag*, *pol*, and *env* ORF translation start sites (∆GPE, mock B), which failed to assemble virions (Figure 3b,c). Strikingly, for all individual virus supernatants a perfect correlation between their potentials to induce ISG expression and the upregulation of CD86 cell surface expression in MDDCs was observed (Figure 3d).

Taken together, these results point to the dependence on cytoplasmic access and enzymatically active reverse transcriptase for PFV-mediated ISG induction and activation of MDDCs.

### 3.4. PFV ISG Induction Does Not Require Reverse Transcription upon Target Cell Entry and Is Not Suppressed by SAMHD1

**Figure 4:**
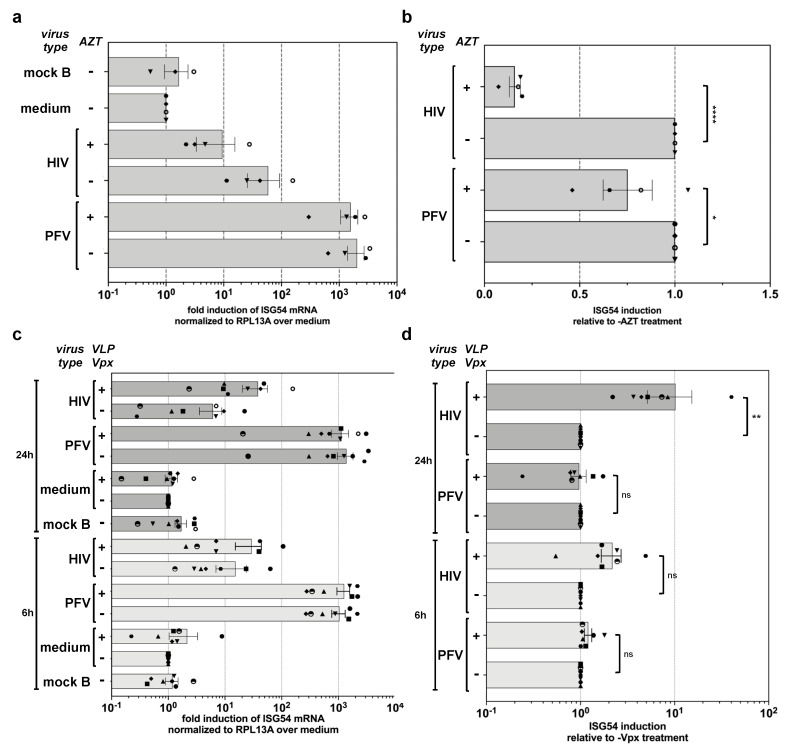
Influence of RTr inhibition and VLP-Vpx treatment on PFV-mediated ISG induction during target-cell entry. (**a**,**b**) MDDCs were incubated with wild type PFV-RCP (MOI 0.25), HIV-1 GFP (MOI 2), and VLP-Vpx or ∆GPE (mock B) mock supernatants in the absence or presence of AZT (100 µM) as indicated. *ISG54* mRNA levels normalized for *RPL13A* mRNA levels were determined by qPCR at 24 h post exposure. (**a**) Mean values ± SEMs, plus individual data points of *ISG54* (*n* = 3–4) induction relative to medium incubated samples are shown. (**b**) Mean values ± SEMs, plus individual data points of *ISG54* (*n* = 4) induction relative to the respective sample incubated with the same virus type without AZT addition are shown. One-way ANOVA with Sidak’s multiple-comparisons test was used to assess significance. (**c**,**d**) MDDCs were incubated with wild type PFV-RCP (MOI 0.25), HIV-1 GFP (MOI 2) supernatants, or ∆GPE (mock B) mock supernatants in the absence or presence of VLP-Vpx as indicated. *ISG54* mRNA levels normalized for *RPL13A* mRNA levels were determined by qPCR at 6 and 24 h post exposure. (**c**) Mean values ± SEMs, plus individual data points, of *ISG54* (*n* = 7) induction relative to medium incubated samples are shown. (**d**) Mean values ± SEMs, plus individual data points, of *ISG54* (*n* = 7) induction relative to the respective sample incubated with the same virus type without VLP-Vpx addition are shown. One-way ANOVA with Sidak’s multiple-comparisons test was used to assess significance. * *p* < 0.05; ** *p* < 0.01; *** *p* < 0.001; **** *p* < 0.0001; ns: not significant (*p* ≥ 0.05).

The above analysis revealed that PFV-RCP mediated induction requires an enzymatically active RT. FVs RTr has been reported to occur both during virus’s assembly and upon target cell uptake and entry [4,5,6]. We therefore aimed to determine whether RTr products already present in PFV-RCP particles or those newly generated during target cell entry, as reported for other retroviruses like HIV-1 [36,37], are the main ISG inductors in myeloid cells. For that purpose, MDDCs were exposed to wild type PFV-RCP and HIV-1 GFP reporter viruses in the presence or absence of AZT, preventing de novo RTr upon target cell entry (Figure S2). Quantification of ISG induction at 24 h p.i. revealed a strong, 5- to 10-fold reduction in *ISG54* induction for HIV-1 GFP exposed samples by AZT treatment (Figure 4a,b). In contrast, AZT treatment diminished the ISG induction potential of PFV-RCP only marginally, a maximum of 2-fold (Figure 4a,b).

Furthermore, we examined whether SIV-Vpx-mediated degradation of endogenous SAMHD1 influences ISG induction mediated by PFV RTr products in MDDCs. MDDCs were exposed to PFV-RCP or HIV-1 GFP supernatants in the presence or absence of SIV-VLPs containing Vpx (Figure S4). Analysis of *ISG54* induction at 6 and 24 h p.i. confirmed the previously reported enhancement of HIV-1 sensing, up to 10-fold at 24 h p.i. (Figure 4c). In contrast, *ISG54* induction levels mediated by PFV-RCPs in MDDCs were not significantly altered by simultaneous Vpx-mediated SAMHD1 inactivation. This is in line with the *ISG56* induction capacity of PFV-RCP in THP-1 SAMHD1 KO cells shown before (Figure 2).

Thus, vDNA or RTr products generated from the encapsidated vRNA genome during virus morphogenesis are the major PFV PAMPs, which are sufficient for efficient sensing of PFV in myeloid cells. Furthermore, unlike HIV, PFV sensing in myeloid cells cannot be enhanced by SIV Vpx pretreatment, arguing against a role of endogenous SAMHD1 in the regulation of PFV sensing.

### 3.5. PFV ISG Induction Requires Reverse Transcription of Full-Length Viral Genomes

**Figure 5:**
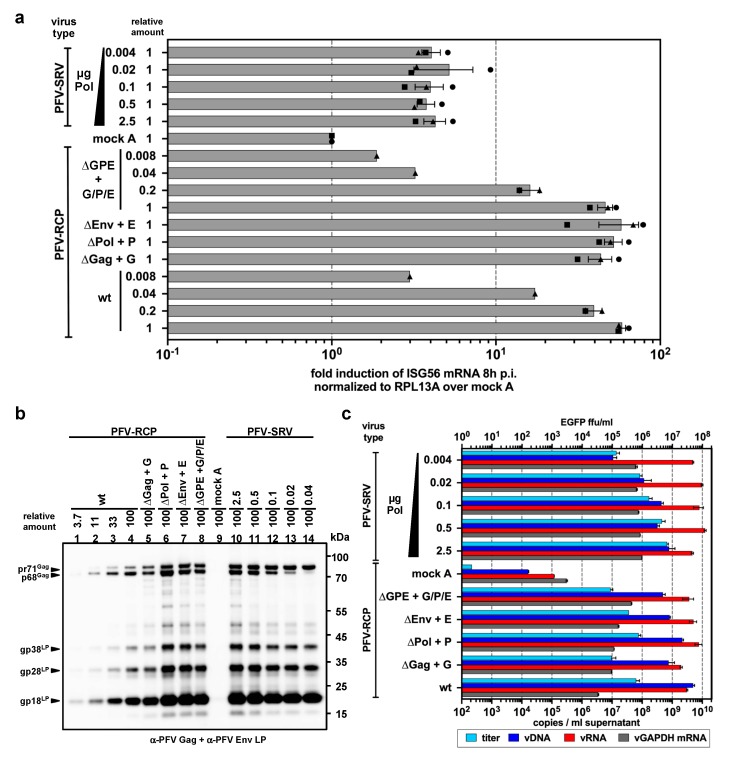
Differential ISG induction profile of single round PFV vector particles harboring full-length or minimal viral genomes. (**a**) PMA-differentiated THP-1 wild type cells were incubated with the indicated relative amounts of wild type PFV-RCP (MOI 0.3) supernatants and variants thereof, or the different PFV-SRV supernatants with variable Pol content (µg Pol packaging plasmid used for supernatant production is indicated; MOI 3 at 2.5 µg Pol) or supernatant from 293T cells transfected with pUC19 (mock A). *ISG56* mRNA levels normalized for *RPL13A* mRNA levels were determined by qPCR at 8 h post exposure. Mean values ± SEMs, plus individual data points of *ISG56* (*n* = 1–3) induction relative to mock A treatment are shown. (**b**,**c**) PFV supernatants characteristics. (**b**) Particle protein composition. Western blot analysis of protein composition of viral particles pelleted by ultracentrifugation of virus supernatants used in panel A employing PFV Gag (α-PFV Gag) and PFV Env LP (α-PFV Env LP) specific polyclonal antisera. The identity of individual protein bands is indicated to the left, the molecular weight to the right. (**c**) Particle nucleic acid composition. Particle-associated nucleic acids extracted from viral particles pelleted by ultracentrifugation of virus supernatants used in panel A were analyzed by qPCR to quantify the particle-associated viral (vDNA: viral DNA; vRNA: viral RNA) and cellular (vGAPDH mRNA) nucleic acid composition. Mean copy numbers ± SDs per mL supernatant determined from duplicates are shown. Viral titers were determined for PFV-SRV supernatants by *EGFP* reporter gene transfer assay on HT1080 cells and for PFV-RCP supernatants by Tas-dependent *EGFP* reporter gene induction assays on HT1080 PLNE cells. Mean titers ± SDs per mL supernatant determined from technical duplicates are shown.

Reports on level of innate sensing of HIV-1 in various target tissues appear to be strongly influenced by the specific type of HIV-1 viruses (full-length genome replication-competent versus single-round versus minimal vector genome) [57,58]. The characterization of the ISG induction profile of the various PFV-RCP mutants described above and the wild type like ISG profile of IFNAR1 THP-1 KO cells suggested that viral spreading in the culture is not required. This and the time course analysis of ISG induction suggest that the ISG response is induced shortly after cytoplasmic entry. Therefore, we also examined whether single-round PFV vector particles containing minimal viral genomic sequences (PFV-SRV) (Figure A1) induce an ISG response in myeloid cells (Figure 5). Surprisingly, whereas exposure of PMA-THP-1 cells to wild type PFV-RCPs lead to a strong *ISG56* induction, *ISG56* induction levels in cells to PFV-SRVs were strongly reduced (Figure 5a).

In addition to their replication capacities there are two major differences between PFV-SRV and PFV-RCP. First, PFV-SRVs, unlike PFV-RCP, package Pol also in a vRNA genome independent manner, which leads to higher particle associated RT levels [41]. Second, the packaged and reverse transcribed minimal vRNA genome of PFV-SRVs lacks large genomic regions and does not encode any viral proteins [45].

To examine whether any of these differences are responsible for the differential ISG induction profile, different PFV-SRV and PFV-RCP supernatants were produced. For PFV-SRV, various supernatants were produced, keeping the amounts of vector genome Gag and Env expression vectors constant but using different amounts of Pol packaging plasmid. This resulted in virus supernatants containing similar physical amounts of PFV particles (Figure 5b), which contained similar amounts of viral and cellular RNA, but differed in their vDNA content (Figure 5c) and specific viral infectivity over a 50 to 100-fold range (Figure 5c). When PMA-THP-1 cells were exposed to identical amounts of these different PFV-SRVs, only a very weak ISG response was detectable, which was not influenced by the vDNA content and was at least 10-fold lower than that of PFV-RCP controls (Figure 5a).

Next, PFV-RCPs variants were used in which the translation of individual (∆Gag, ∆Pol, ∆Env) or all (∆GPE) structural or enzymatic viral ORFs were abolished by point mutagenesis to determine whether the presence of functional ORFs for PFV structural genes in the viral genome is required for efficient innate sensing (Figure A1). Infectious virus supernatants were generated by complementing the individual defective structural functions by using the respective packaging constructs (+G, +P, +E, +G/P/E) also employed for production of PFV-SRVs. RCP virus supernatants contained similar physical amounts of virus particles with similar amounts of vRNA as PFV-SRVs (Figure 5b,c). Mutant PFV-RCP particles contained 2- to 5-fold lower amounts of vDNA and 2- to 8-fold higher amounts of cellular RNA compared to wild type PFV-RCP. Notably, the vDNA of mutant PFV-RCP was similar to that of PFV-SRVs generated with the highest amounts of PFV Pol packaging plasmid (Figure 5c). Strikingly, all mutant PFV-RCP supernatants, including the ∆GPE +G/P/E mutant, did induce an ISG response at wild type level (Figure 5a). When PMA-THP-1 cells were exposed to reducing amounts of PFV-RCP wt and ∆GPE +G/P/E, a dose-dependent decline in the ISG response was observed, with its level correlating well with the amount of vDNA present in the respective supernatants (Figure 5c).

Taken together, these results suggest that the type of PFV RNA genome encapsidated and reverse transcribed is crucial for innate sensing of PFV rather than the amounts of particle-associated Pol and vDNA.

## 4. Discussion

FV infections of natural hosts and zoonotic transmission to humans appear to be efficiently controlled by the immune system. We have only limited knowledge on the immunological mechanisms involved in this process.

Here, we examined the interaction of PFV with the innate immune system in immune cells of the myeloid lineage. Using an in vitro model cell line, THP-1, and primary human MDDC and MDM cultures, we observed an efficient stimulation of the innate immune system, determined as an IRF3-dependent stimulation of ISG expression and IRF3 phosphorylation, by replication-competent PFV generated from proviral expression constructs (PFV-RCP).

Furthermore, PFV innate sensing was neither dependent on viral transactivator Tas-mediated de novo viral transcription nor on viral integrase enzymatic activity. This indicates that productive infections, late steps of the viral replication cycle and virus spreading are not a prerequisite for PFV innate sensing in myeloid cells.

By use of various PFV-RCP mutants we demonstrated that PFV sensing occurs predominantly in the cytoplasm of myeloid cells as fusion-defective PFV-RCPs failed to stimulate ISG expression.

In contrast, efficient ISG induction required an enzymatically active reverse-transcriptase, indicating that vDNA or RTr products generated during reverse transcription are the major PFV PAMPs sensed by the innate immune system. Since FV RTr is observed both late in the replication cycle after capsid assembly and early during host cell entry [4,5,6], we investigated whether vDNA and RTr products already being present in PFV particles or those newly generated during uptake are the major PAMPs. Inhibition of RTr during entry by RT inhibitor led only to a minor reduction in PFV-mediated ISG induction, whereas that of HIV-1 was strongly reduced. This indicates that the vDNA and RTr products already present in PFV particles are sufficient for efficient induction of an innate immune response. However, since we are unable to prevent RTr during PFV assembly and at the same time allow subsequent RTr to take place during virus entry, as AZT incorporation leads to dead-end products, we cannot formally exclude that the latter may contribute to a certain extent to the innate immune response.

In line with the ISG induction potentials of the various PFV-RCP mutants, we observed that inactivation of essential key molecules of cellular DNA-sensing pathways, cGAS and STING, in THP-1 cells, abolished PFV-RCP-mediated innate immune stimulation. In accordance with vDNA and RTr products already present in PFV particles before host cell entry, representing the main PFV PAMPs, inactivation of cellular SAMHD1, either by gene KO in THP-1 cells or VLP-Vpx co-delivery in MDDCs, had only minor negative effects on PFV-RCP mediated ISG induction. This is also in agreement with previous reports of SAMHD1, unlike for lentiviruses, not being a restriction factor for PFV [59].

pDCs were shown by Rua and colleagues to mount an innate immune response as a consequence of TLR7-mediated sensing of PFV RNA [23]. Our results suggest that in myeloid cells, the contribution of vRNA sensing to PFV-mediated innate immune stimulation appears to be negligible. This is underlined by clearly detectable, although slightly reduced ISG induction in THP-1 KO cells having key molecules of cellular RNA-sensing pathway, MAVS or MyD88, inactivated. Furthermore, PFV-mediated ISG induction was almost completely abolished when myeloid cells were incubated with PFV-RCP with enzymatically inactive RT, which did not contain vDNA but harbored similar levels of vRNA as wild type virus.

The most striking finding of this study was the requirement of vDNA and/or RTr products to be derived from full-length vRNA genomes (PFV-RCP) instead of RNA genomes with minimal cis-acting viral sequences of PFV single-round vectors (PFV-SRV) for efficient ISG induction in myeloid cells. Interestingly, in contrast to the minimal genome of single-round vectors (PFV-SRV wt), which had a strongly impaired ISG induction capacity, the RTr of single-round vectors encapsidating a full-length genome containing point mutations (PFV-RCP ∆GPE + G/P/E) led to an ISG induction profile similar to replication-competent, wild type PFV particles (PFV-RCP wt). Our results obtained with various kinds of PFV-SRVs and PFV-RCP mutants can rule out differences in the replication capacity, the encoding of structural proteins, and the particle-associated RT levels or vDNA copy numbers as causative for this difference in innate stimulatory capacity. This underscores that most likely the encapsidated and reverse transcribed full-length genome represents the immunostimulatory component. Currently we can envision several potential underlying mechanisms.

A very attractive but perhaps also the most unlikely explanation might be the presence of immunostimulatory determinants in full-length PFV genomes that are absent in minimal PFV vector genomes. A specific **v**iral **s**timulatory **s**equence **e**lement (vSSE), and/or secondary structure thereof, present only in RTr products of full-length PFV genomes, could potentially be sensed.

Alternatively, the size difference between full-length PFV proviral (11,024 nt) and minimal SRV genomes (4348 nt), and not, or not only, a specific vSSE absent in the latter, may be responsible for or contribute to their differential ISG induction potential. This would fit to reports of cGAS activation based on DNA length and based on long DNA pre-structured by host proteins to strongly stimulate DNA sensing [60,61,62]. HIV-1 minus strand strong stop DNA ((-)sssDNA) was reported to contain short stem-loop structures with flanking unpaired guanosines highly stimulatory for cGAS activity. Notably, full-length PFV-RCP and minimal PFV-SRV genomes are identical up to the translation start of the *gag* ORF, thereby leading to identical PFV (-)sssDNA’s (Figure A1). Therefore, even if PFV (-)sssDNA harbors cGAS stimulatory structures analogous to HIV-1 (-)sssDNA, it cannot be the cause for the differential ISG stimulatory capacity of RTr products of full-length PFV-RCP compared to minimal PFV-SRV genomes.

Finally, we cannot rule out that currently unknown differences in stability, structural integrity, or uncoating of the infecting viral cores containing full-length wild type or point mutation-containing genomes in comparison to minimal vector genomes are causing the difference in the innate response.

Further studies, including a detailed bioinformatic analysis of secondary structure prediction of PFV RTr products and their experimental verification, are required to provide experimental evidence for any of the proposed mechanisms, and combinations thereof, or they may reveal a currently unknown way of innate sensing of FV vDNA or RTr products and may identify additional cellular factors involved in this process.

## 5. Conclusions

To the best of our knowledge, this study is the first to demonstrate that replication-competent FVs can efficiently stimulate an innate immune response in human immune cells of the myeloid lineage. With FV particles known to contain significant amounts of reverse transcribed viral genome, it was not surprising that vDNA and/or RTr intermediates represent the PFV PAMP and cGAS, the main cellular PRR that are responsible for mounting an IRF3-dependent ISG response in this cell type. Elucidation of the underlying mechanism responsible for the differential innate stimulating capacities of full-length and minimal vector genomes in further studies may result in a more detailed characterization of the PFV genomic structures or sequence elements recognized by the host cell’s innate immune system.

## Supplementary Materials

The following are available online at https://www.mdpi.com/1999-4915/11/12/1095/s1. Figure S1: Characterization of MDDC and MDM differentiation status; Figure S2: AZT-mediated inhibition of MDDC transduction by HIV-1 GFP; Figure S3: PFV-mediated IRF3 phosphorylation in MDDCs; Figure S4: Degradation of endogenous SAMHD1 upon VLP-Vpx transduction.

## Appendix A

**Table A1:** Primer–probe set for Taqman qPCR analysis.

Target	Primer/Probe	Sequence [5′-3′]	Cycle Conditions
PFV	fwd	TGCAATTCCAAAGGTGATTC	95 °C, 8 min, 1×
RNA/DNA	rev	TACCTCTTTCCTTTGCCCAT	95 °C, 30 s, 40×
	probe	TCAAGGTGCAGCATTCACTTCTTCAA	59 °C, 30 s, 40×
			72 °C, 90 s, 40×
hGAPDH	fwd	CATCAATGGAAATCCCATCA	95 °C, 8 min, 1×
	rev	GACTCCACGACGTACTCAGC	95 °C, 30 s, 40×
	probe	TCCAGGAGCGAGATCCCTCCA	59 °C, 30 s, 40×
			72 °C, 90 s, 40×
hISG56	fwd	GAGATCATTCACACCTCATTAT	95 °C, 8 min, 1×
	rev	GGAGTGAGTCTTCTGTTCT	95 °C, 30 s, 40×
	probe	TTCCCTCCCTTGCTAACTGCCATT	58 °C, 30 s, 40×
			72 °C, 90 s, 40×
hRPL13A	fwd	CCACCCTGGAGGAGAAGAGG	95 °C, 8 min, 1×
	rev	GAGTCCGTGGGTCTTGAGGA	95 °C, 30 s, 40×
	probe	TCGGCCTGTTTCCGTAGCCTCA	58 °C, 30 s, 40×
			72 °C, 90 s, 40×

**Table A2:** Primer–probe set for SYBR Green qPCR analysis.

Target	Primer/Probe	Sequence [5′-3′]	Cycle Conditions
hISG54	fwd	GGTGGCAGAAGAGGAAGATT	50 °C, 30 min, 1×
	rev	TAGGCCAGTAGGTTGCACAT	95 °C, 15 min, 1×
			94 °C, 15 s, 40×
			56 °C, 45 s, 40×
			72 °C, 45 s, 40×
hRPL13A	fwd	CCTGGAGGAGAAGAGGAAAGAGA	50 °C, 30 min, 1×
	rev	TTGAGGACCTCTGTGTATTTGTCAA	95 °C, 15 min, 1×
			94 °C, 15 s, 40×
			56 °C, 45 s, 40×
			72 °C, 45 s, 40×

**Table A3:** Antibodies used for FACS analysis.

Antibody Specificity	Company	Clone	Volume per Sample in µL	Fluorophore Attached
CD14	BioLegend	M5E2	1	Pacific Blue
CD163	BD Bioscience	GHI/61	2	PE
CD206	BD Bioscience	19.2 (RUO)	2	APC
CD11c	Miltenyi	N418	2	Vio Blue
CD1a	BioLegend	SK9	2	PE
CD16	BioLegend	3G8	2	APC
CD86	BioLegend	IT2.2	1.5	PE
IgG2a, κ; isotype control	BioLegend	MOPC-173	1	Pacific Blue
IgG1, k Isotype control	Beckman Coulter	Clone GHI/61 (RUO)	2	PE
IgG1, k Isotype control	BD Bioscience	MOPC-21	2	APC
IgG2b, isotype control	Miltenyi	IS6-11E5.11	2	Vio Blue
IgG2b, k isotype control	BioLegend	MG2b-57	2 or 1.5	PE
