# Supplementary material for: Insights into Innate Sensing of Prototype Foamy Viruses in Myeloid Cells
Source: Viruses. 2019 Nov 26;11(12):1095. doi: 10.3390/v11121095 (PMC6950106; doi:10.3390/v11121095)
Supplement: Supplementary file 1 [file viruses-11-01095-s001.pdf]

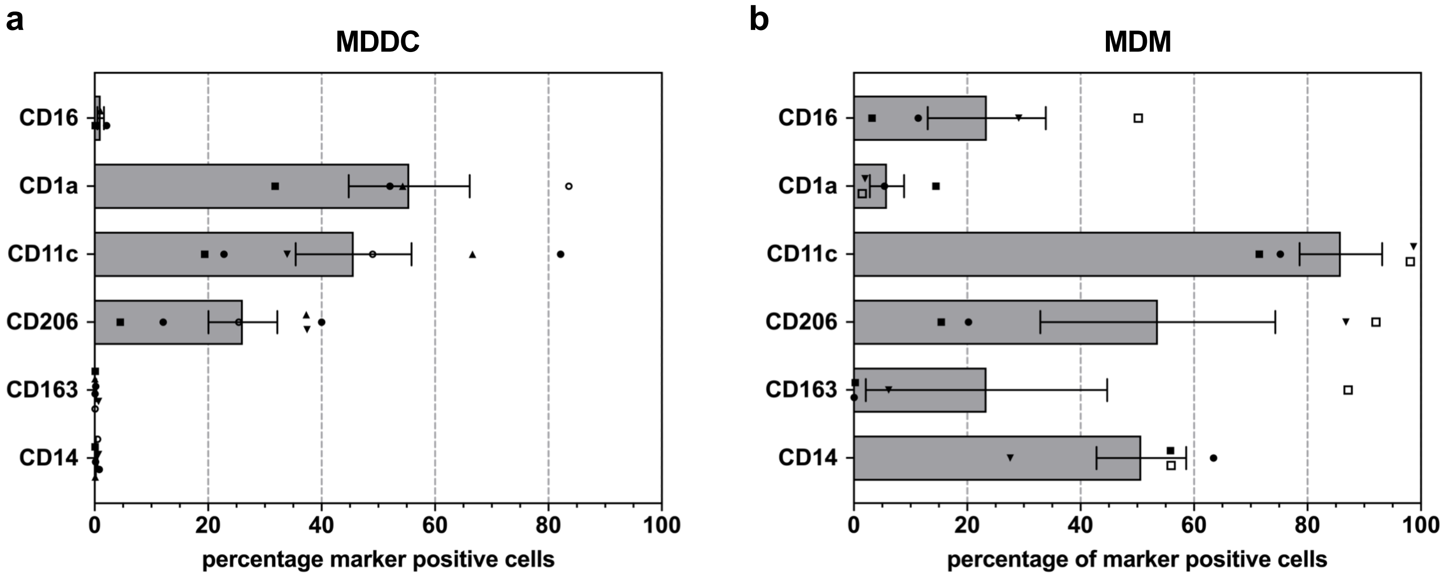

Figure S1:Characterization of MDDC and MDM differentiation status.
Upon five days of differentiation, a) MDDCs and b) MDMs (used in Figure 1, 3, 4) were characterized by determining the expression levels of different surface markers (CD16, CD1a, CD11c, CD206, CD163 and CD14) by immunostaining and flow cytometry analysis. Means ±SEM (n=6 for MDDCs, and n=4 for MDMs) of percentage of marker positive cells, normalized to the corresponding IgG control are shown.

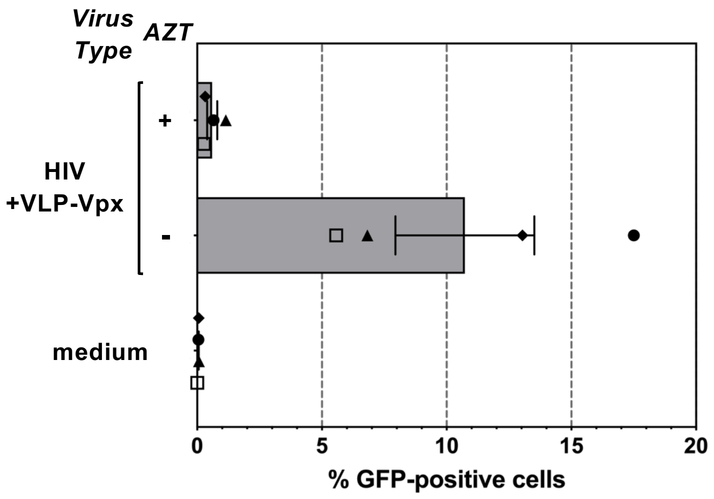

Figure S2: AZT-mediated inhibition of MDDC transduction by HIV-GFP.
MDDCs used in Figure 4a and 4b were incubated with HIV-1 GFP (MOI 2) and VLP-Vpx in the presence or absence of AZT (100 µM). Twenty-four hours post exposure, the percentage of HIV-1 infected GFP-positive cells was determined via flow cytometry and normalized to medium incubated samples. Means ±SEM (n=4) are shown.

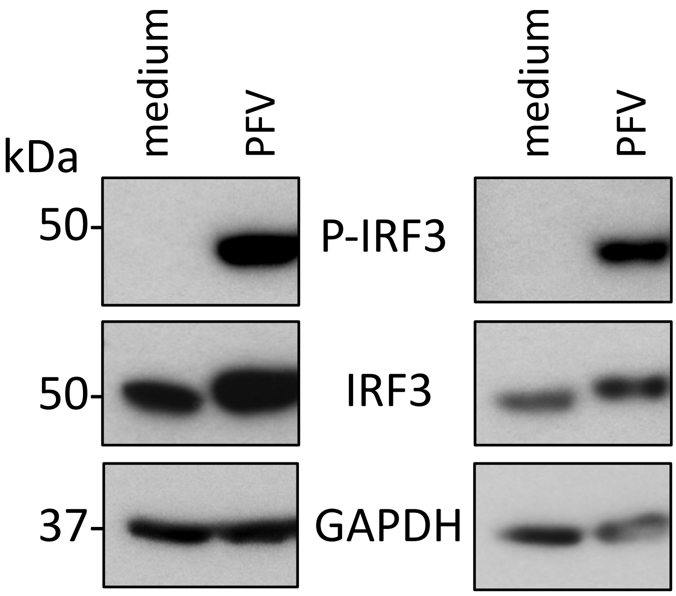

Figure S3: PFV-mediated IRF3 phosphorylation in MDDCs.
Primary human MDDC from two donors were incubated with wild type PFV-RCP (MOI 0.25) supernatant or medium (medium) for 6 h. Whole cell lysates were subjected to immunoblotting and IRF3 phosphorylation was assessed using specific antibodies as indicated.

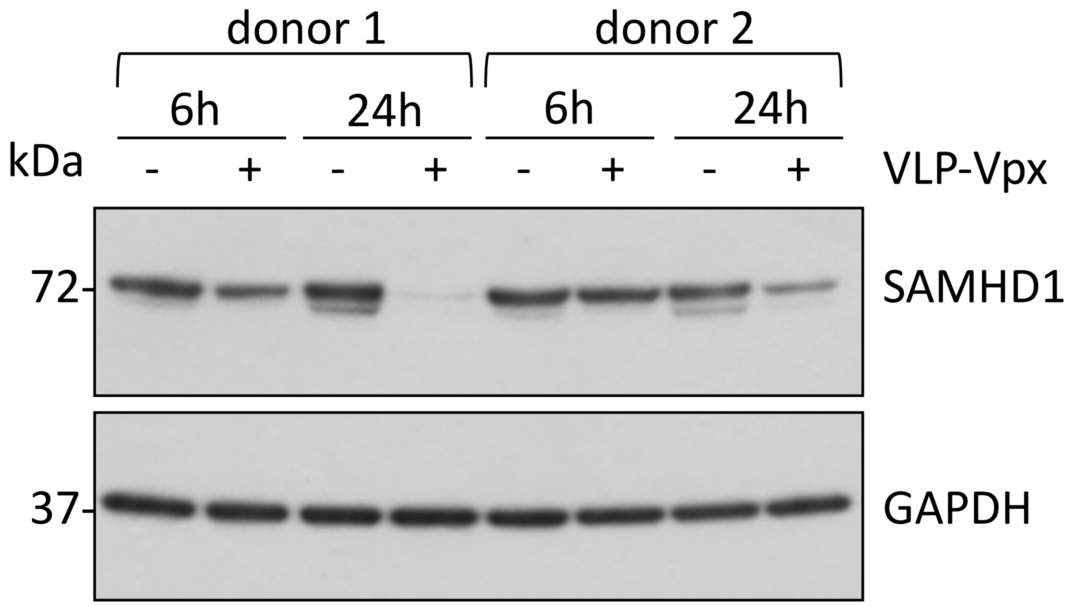

Figure S4: Degradation of endogenous SAMHD1 upon VLP-Vpx transduction.
MDDCs derived from two donors were exposed to the same amount of VLP-Vpx, as used for stimulation experiments (Figure 4). Six and 24 h post exposure, whole cell lysates were subjected to immunoblotting and the level of SAMHD1 degradation was assessed using specific antibodies as indicated.
